# Induction Therapy With Oral Tacrolimus Provides Long‐Term Benefit in Thiopurine‐Naïve Refractory Ulcerative Colitis Patients Despite Low Serum Albumin Levels

**DOI:** 10.1002/jgh3.70139

**Published:** 2025-04-02

**Authors:** Shoko Igawa, Toshihiro Inokuchi, Sakiko Hiraoka, Junki Toyosawa, Yuki Aoyama, Yasushi Yamasaki, Hideaki Kinugasa, Masahiro Takahara, Hiroyuki Okada, Motoyuki Otsuka

**Affiliations:** ^1^ Department of Gastroenterology and Hepatology Okayama University Graduate School of Medicine, Dentistry and Pharmaceutical Sciences Okayama Japan

**Keywords:** biologics therapy, tacrolimus, thiopurine, ulcerative colitis

## Abstract

**Background and Aim:**

Oral tacrolimus is an effective treatment for refractory ulcerative colitis (UC). However, tacrolimus is underutilized because of the difficulties in transitioning to subsequent maintenance therapy and concerns about adverse events.

**Methods:**

We evaluated the clinical outcomes, adverse events, and accumulated medication costs in consecutive 72 UC patients treated with tacrolimus.

**Results:**

Fifty‐five (76%) patients with pancolitis and 43 (60%) patients with acute severe disease were entered. Fifty‐four (75%) achieved clinical remission 8 weeks after starting tacrolimus. At the last visit, 62 (86%) patients had colectomy‐free remission, and 55 (76%) patients had corticosteroid‐free remission. Eighteen (25%) patients maintained remission without additional treatment after tacrolimus discontinuation. Patients with continuous remission had a significantly lower history of thiopurine use and lower serum albumin levels at the induction of tacrolimus than patients with failure to induce or maintain remission. No severe adverse events due to tacrolimus treatment were observed. The accumulated medication costs over 3 years in patients with continuous remission after the start of tacrolimus were lower than those in patients with induction and maintenance of infliximab (*p* < 0.001).

**Conclusions:**

Tacrolimus could have an irreplaceable role in the era of biologic therapies, especially for refractory UC patients with thiopurine‐naïve and low serum albumin levels.

**Abbreviations**
5‐ASA: 5‐aminosalicylic acid
CRP: C‐reactive protein
CyA: cyclosporine A
IBD: inflammatory bowel disease
IFX: infliximab
JAK: Janus kinase
NUDT: nucleoside diphosphate‐linked moiety X‐type motif
Tac: oral tacrolimus
TNF: tumor necrosis factor
UC: ulcerative colitis

## 1 Introduction

Ulcerative colitis (UC) is a chronic inflammatory bowel disease (IBD) that is characterized by frequent relapse and remission. Some patients have refractory disease courses with steroid dependence or resistance and often have difficulties in medical treatment. Recently, treatment options for UC have increased with the introduction of agents, such as biologics and Janus kinase (JAK) inhibitors. In Japan, infliximab (IFX), adalimumab, and golimumab were approved in June 2010, June 2013, and March 2017, respectively. Subsequently, vedolizumab, ustekinumab, and JAK inhibitors were approved in 2018 or later. As these biologics and JAK inhibitors are easy to continue from the induction of remission to maintenance without trough adjustment, these therapies are becoming mainstream for the treatment of refractory UC. However, some patients, especially those with severe activity and low serum albumin levels, may require colectomy because of the low efficacy of biologic therapies [1]. In addition, the high cost of medications is a serious social problem. This is due to the lack of criteria for the discontinuation of these drugs.

Oral tacrolimus (Tac) is a calcineurin inhibitor that has been used effectively in refractory UC patients with moderate‐to‐severe activity [2, 3, 4]. Tac shows a stable effect after oral administration twice daily. A randomized controlled study demonstrated the efficacy and safety of Tac in inducing the remission of refractory UC [2]. In particular, patients with acute severe UC are often rescued, and remission is induced with Tac [2, 3, 4]. However, due to concerns about the side effects caused by prolonged administration, tacrolimus is regarded as a specialized therapy for the induction of remission. After the induction of remission by Tac, the reduction or discontinuation of Tac and a shift to maintenance therapy, such as 5‐ASA or thiopurines, should be considered. Although relapse of the disease course sometimes occurs on switching drugs, this may be, in a paradoxical way, a favorable feature that distinguishes Tac from biologic therapies, the discontinuation of which is rarely actively attempted [5, 6]. Oshima et al. reported the effectiveness of tacrolimus in a multicenter study. However, the long‐term outcomes were not assessed, and its efficacy and safety remain unclear [7].

IFX, currently the most commonly used biologic for patients with severe refractory UC, is acceptable for long‐term administration if it is effective. Many studies comparing IFX and Tac for severe refractory UC have been reported. Most studies have shown that IFX and Tac have equivalent efficacy [8], but some have described IFX as a more effective and durable therapy than Tac [9]. In contrast, other reports have suggested that hypoalbuminemia predicts IFX failure in acute severe UC [1]. Furthermore, in a recent study, Brandse et al. reported an association between a lack of response to IFX and leakage of IFX into feces in patients with severe UC [10]. Patients with low serum albumin levels had high fecal IFX concentrations but also low serum IFX concentrations, indicating that patients with low serum albumin levels may not effectively respond to IFX therapy. In addition, biosimilars, which are less expensive than the original drugs, are incorporated, but biologic therapy has a significant impact on healthcare costs [11, 12, 13, 14].

In this study, we investigated the clinical role of Tac in patients with refractory UC in the era of biologic therapies by retrospectively examining the long‐term clinical courses of patients with UC treated with Tac.

## 2 Methods

### 2.1 Patients

Data on all consecutive patients with moderate‐to‐severe UC who were treated with Tac at Okayama University Hospital from April 2009 to September 2019 were retrospectively analyzed. The exclusion criteria were a follow‐up period of less than 3 years and discontinuation of Tac within 1 week of starting Tac. The characteristics and clinical courses of eligible patients were obtained from medical charts, and if the patients had received Tac two or more times, the initial administration episode alone was included in this analysis. Disease severity was adapted from Truelove and Witts' criteria [15]. In particular, acute severe colitis was defined as ≥ 6 bloody stools daily with evidence of systemic toxicity (fever, tachycardia, anemia, or an elevated erythrocyte sedimentation rate) [16]. Endoscopic severity was estimated according to the Mayo Endoscopic Subscore before the start of Tac. If patients had severe abdominal pain, such as fulminant conditions, we prioritized their safety and did not perform colonoscopy before Tac therapy.

### 2.2 Administration of Tac and Remission Maintenance Therapy

Generally, Tac is administered to UC patients according to the clinical practice guidelines [17]. Tac was administered to patients with moderate‐to‐severe UC who did not respond to corticosteroids. In particular, patients with severe or fulminant UC were administered Tac therapy as a priority over other therapies in collaboration with a specialized IBD surgeon.

The details of the administration of Tac therapy have been described previously [18]. In brief, the initial oral dose of Tac was approximately 0.1 mg/kg/day twice daily. The whole blood trough concentration of Tac was measured at our hospital once every 2–3 days by an affinity column‐mediated immunoassay. The target whole blood trough concentration was 10–15 ng/mL (high trough concentration), and dose adjustments were performed to achieve the target concentration as soon as possible within 1 week. After the blood trough concentration exceeded 10 ng/mL, a high trough concentration was maintained for 2–3 weeks. All responding patients were followed up with tapered blood trough levels of 5–10 ng/mL (low trough concentration) for approximately 3–6 months and monitored for adverse events. If patients had adverse events, such as renal disorders, we immediately decreased the blood trough concentration of Tac until function recovered. Adverse events could not be controlled despite a decrease in Tac, and Tac was completely discontinued. During the adjustment period of Tac, corticosteroids were tapered by reducing the dose by 5–10 mg/week and discontinued completely if possible.

In principle, thiopurine (azathioprine/6‐mercaptopurine) was administered as maintenance therapy. After the tacrolimus concentration entered the low‐trough phase, we started the administration of thiopurine, and tacrolimus was discontinued. The dose of thiopurines was adapted to achieve white blood cell counts between 3000 and 6000. Genotyping of the nucleoside diphosphate‐linked moiety X‐type motif (NUDT) 15 codon 139 predicted acute severe thiopurine‐induced leukopenia and alopecia in Asian patients [19]. Therefore, genotyping of NUDT15 codon 139 was performed prior to thiopurine administration in April 2016 [20].

### 2.3 Evaluating the Disease Course

We evaluated the short‐ and long‐term clinical courses of UC patients treated with Tac. The short‐term efficacy of Tac was evaluated based on the remission rate within 8 weeks after starting Tac. Clinical remission was defined as a Mayo stool frequency subscore of 0 or 1 (0, normal number for this patient; 1, 1–2 stools more than normal) and a Mayo rectal bleeding subscore of 0 (0, no blood seen) [21]. The evaluation of long‐term outcomes included the continuous remission rate and colectomy‐free rate. Relapse was defined as a flare‐up of symptoms that required additional treatment, such as corticosteroids, tacrolimus, biologics, JAK inhibitors, or colectomy.

### 2.4 The Comparison of the Cumulative Costs for Tac and Other Treatments

To elucidate the economic efficiency of Tac, we reviewed the medical records to calculate the cumulative drug costs that all patients needed over a three‐year period, when the three‐year period was divided into four terms, and the average cost for both groups in each period was calculated based on their prescription records. We compared the costs of a group that was able to induce remission with Tac and maintain remission with 5‐aminosalicylic acid (5‐ASA), thiopurine, or both with those who were not able to induce or maintain remission. In addition, the estimated cost of IFX treatment, which is the first and leading biologic treatment commonly administered to patients with moderate to severe UC in Japan along with Tac therapy, was adopted as a control and similar to the costs of Tac. Calculations assumed that IFX treatment would be administered at a dose of 300 mg per infusion at weeks 0, 2, and 6, and then every 8 weeks for maintenance therapy based on the average body weight in this study. The drug prices are shown in Table S1.

### 2.5 Statistical Analyses

Patient characteristics were compared using the chi‐square test, Fisher's exact test, and Wilcoxon's test, as appropriate. Continuous variables were reported as median values with ranges and were compared using paired *t*‐tests. Continuous remission and colectomy‐free rates were assessed using the Kaplan–Meier method. The cumulative costs of Tac‐treated patients were compared with those of IFX‐treated patients, which were calculated using simulated fixed values, using the Wilcoxon signed‐rank test. Statistical analyses were conducted using the JMP software program version 16.0 pro for Windows (SAS Institute Inc., Cary, NC, USA). Statistical significance was set at *p*‐value < 0.05.

### 2.6 Ethics Declarations

This study was approved by the Institutional Review Board of Okayama University Graduate School of Medicine (IRB number: 2201‐003) conducted in accordance with the Declaration of Helsinki. All research was performed in accordance with relevant guidelines/regulations. Informed consent was obtained from each patient and/or their legal guardians.

## 3 Results

### 3.1 Patient Characteristics

Eighty‐one patients with UC were treated with Tac between 2009 and 2019 at our hospital. Of these, we excluded six patients who were followed up for less than 3 years and three patients who discontinued Tac within a week because of adverse events. All three patients could not take any tablets at all due to nausea, regardless of the severity of their disease. The remaining 72 UC patients were enrolled in this study. Patient characteristics are shown in Table 1. We used Tac only for remission induction; therefore, Tac was discontinued in all patients. The median (IQR) period of Tac administration was 214 (120.3–334.8) days. Forty (56%) men and 32 (44%) women had a median age of 43 years (range 28.3–54.8 years old) at the start of Tac, and their median duration of disease was 18 months (range 4.3–84 months). Fifty‐five (76%) patients had pancolitis, and 43 (60%) patients had acute severe disease. Sixty‐seven (93%) patients used 5‐ASA, 66 (92%) used corticosteroids, and 30 (42%) used thiopurine (azathioprine or 6‐mercaptopurine). While 20 (28%) patients had a history of failure of anti‐tumor necrosis factor (TNF) α agents before starting Tac, all patients discontinued anti‐TNFα agents just before initiation of Tac.

**TABLE 1:** Characteristics of the study patients.

Patients	*n* = 72
Gender	
Male/female	40 (56%)/32 (44%)
Median (IQR) age at initiation of Tac, years	43 (28.3–54.8)
Median (IQR) duration of disease at initiation of Tac, months	18 (4.3–84)
Location of disease	
Pancolitis/left‐sided	55 (76%)/17 (24%)
Disease severity	
Acute severe/non‐acute severe	43 (60%)/29 (40%)
Response of corticosteroid	
Naïve/dependent/refractory	6 (8%)/26 (36%)/40 (56%)
Previous therapy	
5‐aminosalicylic acid	67 (93%)
Systematic corticosteroid	66 (92%)
Thiopurine	30 (42%)
Anti‐TNFα agents	20 (28%)
Laboratory data at initiation of Tac, median (IQR)	
Hemoglobin (g/dL)	10.8 (9.63–12.6)
Platelet count (×10^4^/μL)	35.3 (27.4–47.3)
Albumin (g/dL)	2.85 (2.43–3.3)
C‐reactive protein (mg/dL)	2.23 (0.64–6.27)
Mayo endoscopic score at initiation of Tac^a^	
2/3	18 (28%)/47 (72%)
Median (IQR) period for administration of Tac, days	214 (120.3–334.8)
Median (IQR) follow‐up period from initiation of Tac, years	7.55 (4.89–10.5)

Abbreviations: IQR, interquartile range; Tac, tacrolimus; TNF, tumor necrosis factor.

^a^ Colonoscopy was performed in 65 patients.

### 3.2 Short‐Term Outcomes in Patients Treated With Tac

**FIGURE 1:**
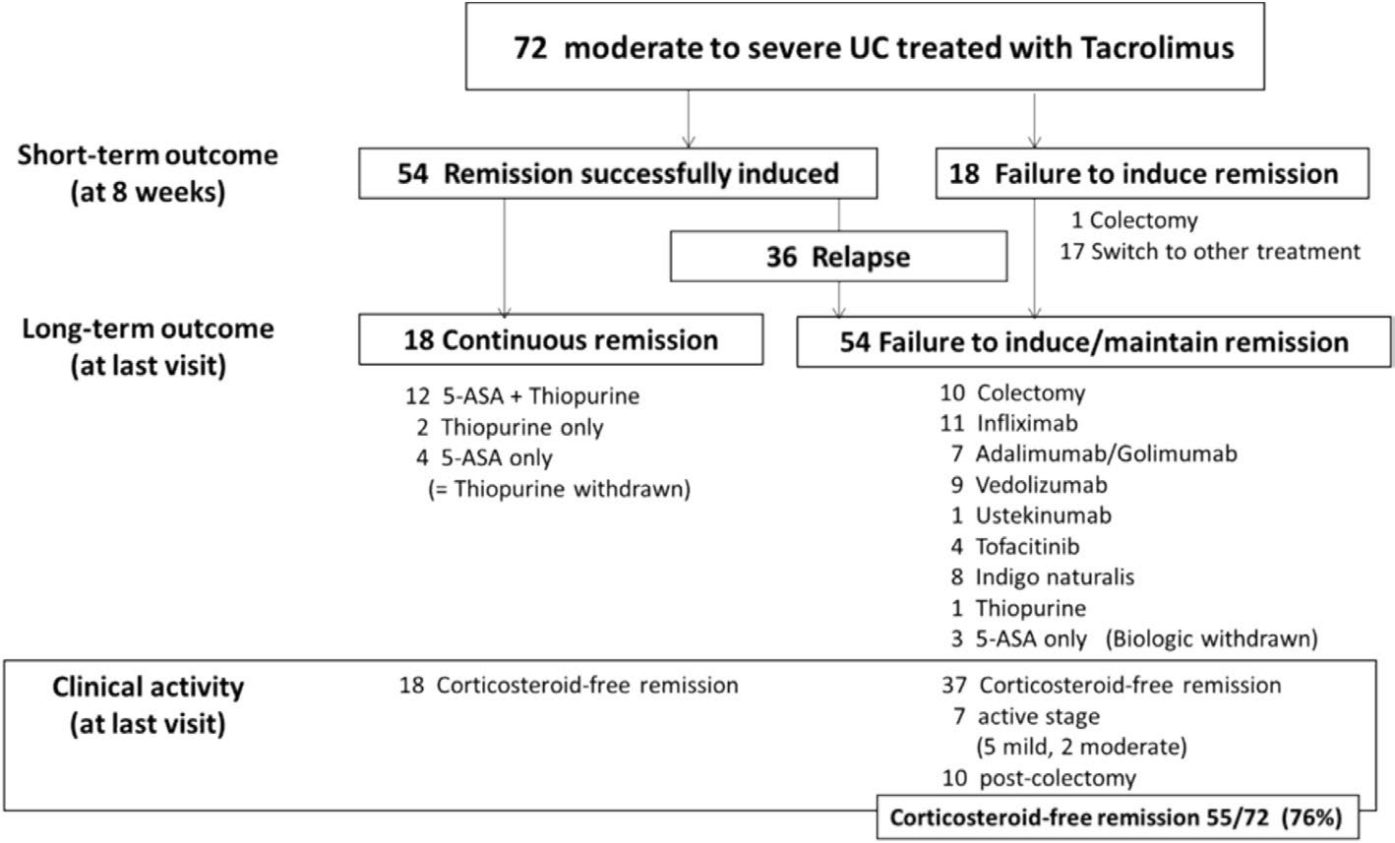
Short‐ and long‐term clinical courses of patients treated with tacrolimus. A flowchart of the clinical courses in patients treated with tacrolimus is shown. Upper part: Short‐term clinical courses within 8 weeks. Middle part: Treatment details at the final visit. Lower part: Clinical activity and steroid‐free remission rate at the final visit.

Although 54 (75%) of 72 patients achieved clinical remission within 8 weeks, 18 (25%) failed to meet the criteria of remission with Tac and received other treatments, including colectomy (Figure 1, upper part). Significantly more patients who failed to induce remission had prior treatment with thiopurines and/or anti‐TNFα agents than those with successful remission induction (thiopurines: 17 [31%] vs. 13 [72%], *p* = 0.0048; anti‐TNFα agents: 10 [19%] vs. 10 [56%], *p* = 0.005) (Table 2). Serum albumin levels and serum hemoglobin levels when Tac was induced were significantly lower in patients with successfully induced remission than in patients with failure to induce remission (albumin: 2.7 [2.4–3.2] g/dL vs. 3.3 [2.9–3.7] g/dL, *p* = 0.014; hemoglobin: 10.6 [9.4–12.2] g/dL vs. 11.9 [10.1–13.1] g/dL, *p* = 0.044) (Table 2). In addition, patients with a short disease course and acute severe UC tended to have a high efficacy rate in this study (*p* = 0.057 and 0.053, respectively) (Table 2).

**TABLE 2:** Comparison of clinical factors between patients with and without remission Induction in the short term.

	Remission successfully induced (*n* = 54)	Failure to induce remission (*n* = 18)	*p*
Gender (male)	28 (52%)	12 (67%)	0.41
Median (IQR) age at starting Tac, years	41.5 (27.8–54)	45 (33–63.5)	0.26
Median (IQR) duration of disease at initiation of Tac, months	13 (3.0–72)	55 (13.8–94.8)	0.057
Location of disease (pancolitis)	42 (78%)	13 (72%)	0.75
Disease severity (acute severe)	36 (67%)	7 (39%)	0.053
Previous therapy			
5‐aminosalicylic acid	49 (91%)	18 (100%)	0.32
Systematic corticosteroid	48 (89%)	18 (100%)	0.33
Thiopurine	17 (31%)	13 (72%)	0.0048
Anti‐TNFα agents	10 (19%)	10 (56%)	0.005
Laboratory data at initiation of Tac			
Hemoglobin (g/dL), median (IQR)	10.6 (9.4–12.2)	11.9 (10.1–13.1)	0.044
Platelet count (×10^4^/μL), median (IQR)	35.3 (28.3–49.0)	33.6 (15.0–42.4)	0.18
Albumin (g/dL), median (IQR)	2.7 (2.4–3.2)	3.3 (2.9–3.7)	0.014
C‐reactive protein (mg/dL), median (IQR)	2.9 (0.70–7.37)	1.61 (0.56–2.5)	0.14
Mayo endoscopic score 3^a^	34 (71%)	13 (76%)	0.76
Median (IQR) period for administration of Tac, days	215 (157–369)	190 (50–330)	0.12

Abbreviations: IQR, interquartile range; Tac, tacrolimus; TNF, tumor necrosis factor.

^a^ Colonoscopy was performed in 65 patients.

### 3.3 Long‐Term Clinical Courses of the Patients Treated With Tac

**FIGURE 2:**
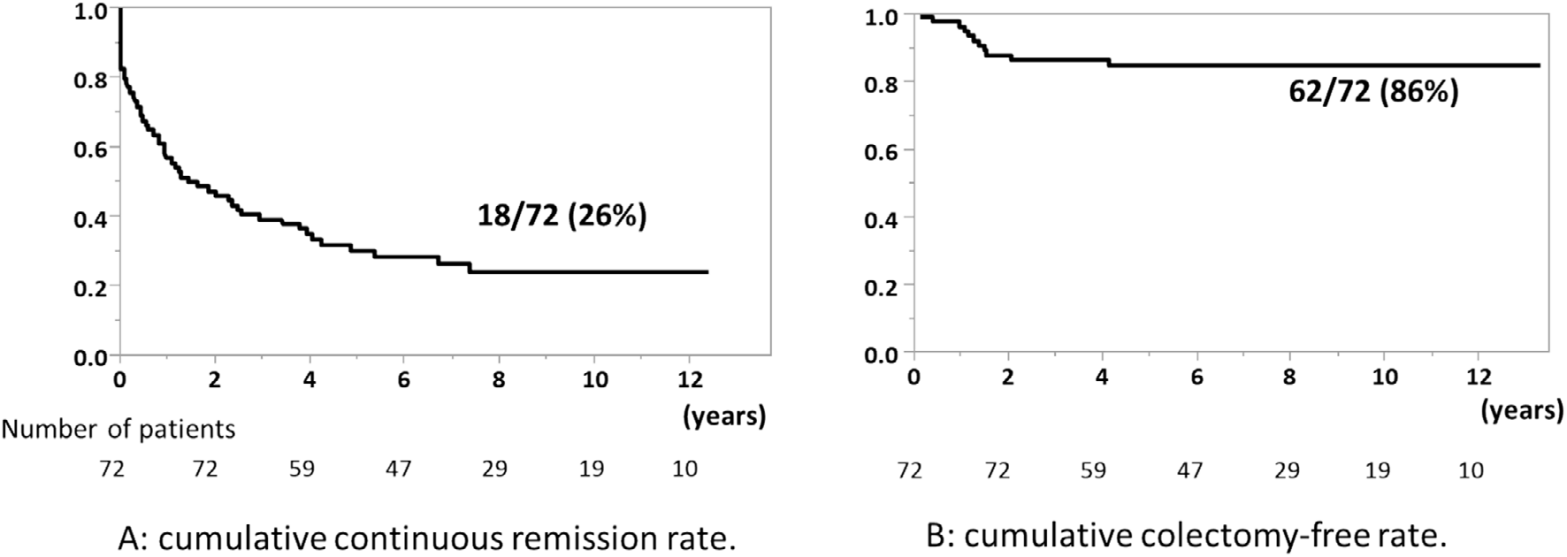
Results of a Kaplan–Meier analysis of the cumulative colectomy rate and continuous remission rate in the long‐term clinical course. (A) Cumulative continuous remission rate. (B) Cumulative colectomy rate.

Eighteen (33%) of 54 patients with successful remission induction within 8 weeks after the start of Tac had maintained continuous remission status when the follow‐up period ended, and all of them needed only 5‐ASA and/or thiopurine for the maintenance of remission (shown in Figure 1, middle and lower part). However, in the other patients, the disease relapsed during the follow‐up period. The cumulative continuous remission rate was 26% (Figure 2A). Significantly more patients with continuous remission had no history of thiopurine use before the start of Tac than patients with failure to induce or maintain remission (patients with continuous remission: 1 [6%] vs. patients with failure to induce or maintain remission; 29 [54%], *p* = 0.0003). Albumin levels were significantly lower in patients with continuous remission at the time of Tac induction than in patients with failure to induce or maintain remission (2.4 [2.2–3.1] g/dL vs. 3.0 [2.6–3.5] g/dL, *p* = 0.013) (Table 3).

**TABLE 3:** Comparison of clinical factors between patients with continuous remission and with failure to induce/maintain remission in the long term.

	Continuous remission^a^ (*n* = 18)	Failure to induce/maintain remission (*n* = 54)	*p*
Gender (male)	9 (50%)	31 (57%)	0.60
Median (IQR) age at starting tacrolimus, years	43.5 (37–58.3)	42 (26.3–54.3)	0.26
Median (IQR) duration of disease at starting tacrolimus, months	6.5 (2.8–59)	27.5 (7.5–94)	0.12
Location of disease (pancolitis)	12 (67%)	43 (80%)	0.34
Disease severity (acute severe)	14 (78%)	29 (54%)	0.097
Previous therapies			
5‐aminosalicylic acid	15 (83%)	52 (96%)	0.10
Systematic corticosteroid	16 (89%)	50 (93%)	0.63
Thiopurine	1 (6%)	29 (54%)	0.0003
Anti‐TNFα agents	3 (17%)	17 (31%)	0.22
Laboratory data at initiation of tacrolimus			
Hemoglobin (g/dL), median (IQR)	10.7 (9.4–12.0)	10.9 (9.7–12.6)	0.47
Platelet count (×10^4^/μL), median (IQR)	38.3 (32.9–48.1)	34.9 (26.6–47.0)	0.35
Albumin (g/dL), median (IQR)	2.4 (2.2–3.1)	3.0 (2.6–3.5)	0.013
C‐reactive protein (mg/dL), median (IQR)	6.86 (1.39–9.60)	1.83 (0.59–5.47)	0.041
Mayo endoscopic score 3^b^	11 (73%)	36 (72%)	1.00
Median (IQR) period for administration of tacrolimus, days	215 (175–303)	211 (111–337)	0.59
Median (IQR) follow‐up period from initiation of tacrolimus, years	6.90 (5.42–9.97)	7.74 (4.65–10.8)	0.79

Abbreviations: IQR, interquartile range; TNF, tumor necrosis factor.

^a^ Continuous remission was defined in cases without escalation of treatment, including corticosteroids, biologics, JAK inhibitors, or colectomy, after achieving clinical remission.

^b^ Colonoscopy was performed in 65 patients.

In contrast, among the 18 patients who failed to induce remission within 8 weeks after the start of Tac, 17 switched to other treatments, and one underwent colectomy. Of the 54 patients who failed to induce or maintain remission, 10 required colectomies, 18 used anti‐TNFα agents (11 IFX and 7 adalimumab/golimumab), 9 used vedolizumab, 1 used ustekinumab, 4 used tofacitinib, 8 used indigo naturalis, 1 received intensification of thiopurine, and 3 used 5‐ASA only (withdrawal of biologics for continuous remission).

All 72 patients discontinued Tac therapy during their clinical course. At the end of the follow‐up period (median follow‐up period 7.55 [4.89–10.5] years), 55 (76%) patients were in steroid‐free remission without colectomy. The cumulative colectomy‐free rate was 86% during the follow‐up period (Figure 2B).

### 3.4 Adverse Events Related to Tac

The details and frequencies of adverse events extracted from the blood test results and medical chart entries are shown in Table 4. In particular, hypomagnesemia, the most common adverse event in this study, did not cause severe symptoms (e.g., arrhythmia or tremor) in patients without any treatment. Renal disorders appeared in 16 patients (22%), and all of them improved rapidly by reducing the dose of Tac. All of these adverse events were reversible by reducing or discontinuing the dose of Tac, and no specific interventions were required for their management.

**TABLE 4:** Adverse events that occurred during tacrolimus therapy.

Adverse events	Cases (%)
Hypomagnesaemia (< 1.2 mg/dL of serum magnesium during tacrolimus therapy)	20 (28%)
Renal disorders (> 1.5 times of serum creatinine at starting tacrolimus)	16 (22%)
Digestive symptoms	17 (24%)
Tremor	11 (15%)
Headache	6 (8%)
Infections	6 (8%)
Abnormal value of β‐D glucan	2 (3%)

### 3.5 The Comparison of the Cumulative Costs for Tac Treatments and Other Treatments

**FIGURE 3:**
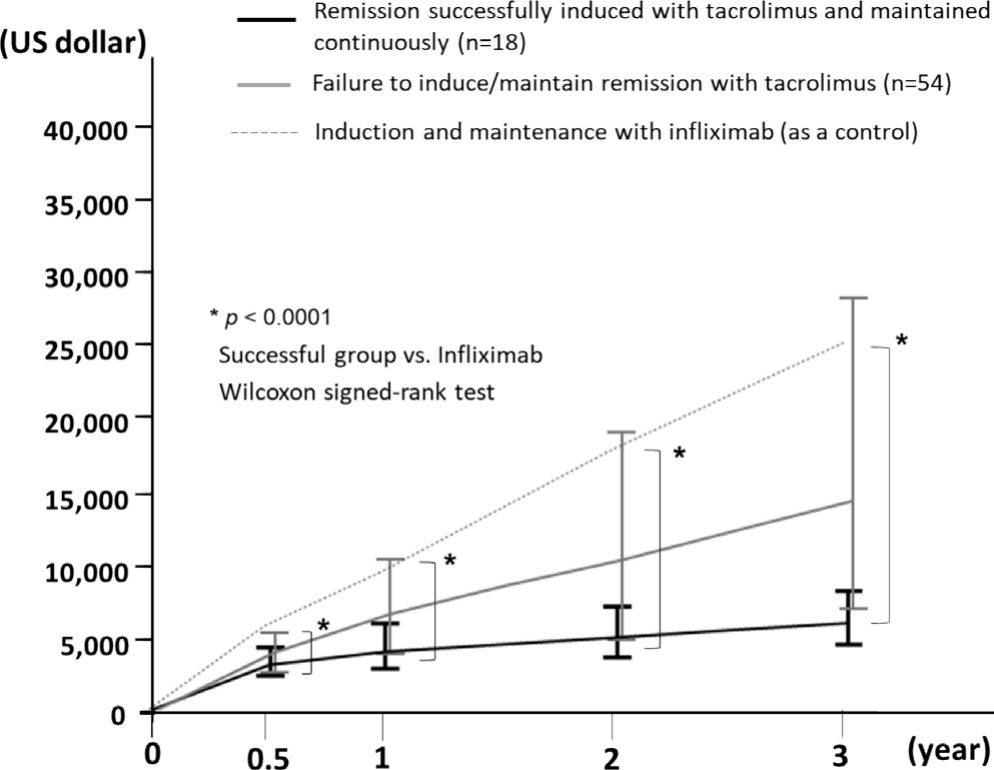
Cumulative cost over 3 years. The median cumulative drug costs over 3 years with Tac and the control case with IFX. The cumulative cost in patients with successful remission induction with Tac was significantly lower than those of the control patients with IFX at all time points (0–0.5, 0–1, 0–2, and 0–3 years; *p* < 0.0001, Wilcoxon signed‐rank test). For the group with Tac treatment, the upper horizontal line shows the 75th percentile and the lower horizontal line shows the 25th percentile. The graph line connects the median.

We calculated the cost of IFX by referring to the average weight of 60 kg in this study and compared it with the cost of Tac. The median cumulative drug costs over 3 years in 72 patients with Tac were approximately $16,000 lower in the control cases with IFX, and this difference was statistically significant (Tac: $8870 vs. IFX: $24 900; *p* < 0.0001, Wilcoxon signed‐rank test). In addition, the cumulative cost in 18 patients with successful remission induction with Tac was significantly lower than those of the control patients with IFX at all time points (0–0.5, 0–1, 0–2, and 0–3 years; *p* < 0.0001, Wilcoxon signed‐rank test) (Figure 3). Similarly, when considering the generic and biosimilar costs of all drugs, including IFX biosimilar, a substantial difference was observed between Tac and IFX biosimilar (*p* < 0.05, Wilcoxon signed‐rank test), with their three‐year costs being $5140 and $9850, respectively (Figure S1).

## 4 Discussion

In this study, we evaluated the short‐ and long‐term clinical courses of patients with moderate‐to‐severe UC who were treated with Tac. Tac had a high remission induction rate (75%) after 8 weeks. Remission induction could be expected even in refractory UC patients with low serum albumin levels due to severe mucosal inflammation, which is an unfavorable factor for IFX therapy [1]. Although the subsequent relapse rate was relatively high, one‐fourth of the patients maintained continuous remission without the use of biologics or JAK inhibitors. Interestingly, patients with continuous remission had lower serum albumin levels and higher C‐reactive protein (CRP) levels at the initiation of Tac than those who failed to induce or maintain remission. In addition, the estimated cumulative costs for the therapeutics were lower when Tac was used as an induction therapy than when IFX was used.

Compared to previous reports, the rate of successful induction of disease remission in this study was higher [3, 22, 23, 24, 25]. One possible reason is that the trough levels of Tac in patients' sera could be immediately measured even on weekends or nights in our hospital, which made it possible to closely monitor the trough levels of Tac in sera. In addition, the dose of Tac was always decided after consultation with physicians experienced in trough control of Tac, resulting in a median of 4.5 days to reach the target trough level (> 10 ng/mL) of Tac in this study.

Based on the present findings, Tac‐preferred patients had hypoalbuminemia and anemia. Tac, a middle molecular weight compound, is an oral immunosuppressive macrolide that potently inhibits T helper lymphocyte activation. Therefore, Tac was able to maintain sufficient drug concentrations for efficacy even in patients with hypoalbuminemia and anemia, in contrast to IFX, a high‐molecular‐weight compound that is lost via feces in severe cases [10]. Tac was also effective for those who had not previously been treated with thiopurines or anti‐TNF‐α agents. This was comparable to a previous finding, in which thiopurine‐naïve patients achieved remission more frequently after Tac therapy than those with a history of thiopurine usage [26]. However, Tac therapy was effective in patients with a short disease course and acute severe UC in this study. It has been reported that the cytokine profile of UC is Th1‐dominant in the early stages of onset and gradually changes to Th2‐dominant during the disease course [27]. Tac is known to significantly suppress Th1 cells rather than Th2 cells [28]. It is theoretically consistent that Tac is highly effective for early onset UC with Th1 dominance and insufficiently effective in cases of UC with a long duration since the onset of the disease. Cyclosporine A (CyA), another calcineurin inhibitor, has been reported to be effective in ASUC [29, 30]. However, no direct comparisons have been made between Tac and CyA. Meta‐analyses have shown that IFX, CyA, and Tac have comparable efficacy, with no significant differences among them, suggesting that all three may be viable treatment options for ASUC [30].

A recent study reported that a high dose (10 mg three times daily) tofacitinib with concomitant intravenous corticosteroids may be an effective induction strategy in biologically experienced patients hospitalized with acute severe UC [31]. JAK inhibitors, which are small‐molecule drugs, have the potential to be saviors for biologics‐refractory UC with hypoalbuminemia, but the accumulation of more clinical data is needed to confirm their efficacy and safety.

Regarding adverse events, several previous reports showed that patients treated with Tac had a higher frequency of adverse events than patients treated with IFX [9, 32]. Several adverse events, such as hypomagnesemia (28%), renal function disorders (22%), digestive symptoms (24%), and infections (8%), were observed in this study. Because of the retrospective study design, mild adverse events such as mild nausea and short‐term tremors may not have been fully documented. Therefore, the overall frequency of adverse events may have been underestimated. In addition, neoplastic lesions were detected in three patients after Tac therapy during the follow‐up period: early‐stage lung cancer (male in his 80s with a history of smoking), early‐stage breast cancer (female in her 60s), and pancreatic neuroendocrine tumor G1 (female in her 40s). All patients underwent surgery and had no recurrence. Although it is difficult to completely exclude the influence of Tac on the occurrence of these neoplasms, it is not necessary to avoid the use of Tac, considering the short‐term exposure to Tac compared to liver transplantation and the low incidence rate [33]. Additionally, no severe adverse events were observed even during the period of concomitant use of AZA and Tac, indicating that the risk of concomitant use of Tac and AZA was very low.

The increasing cost of care due to biologic therapy is a problem [11, 12, 13, 14]. Targownik et al. reported that the annual medical costs for IBD patients nearly tripled in the decade between 2005 and 2015, which was mainly due to the spread of IFX in biologic therapy [13]. In this study, the actual cost of pharmacotherapy for patients with moderate‐to‐severe UC was calculated based on prescription records, and a significant difference was found between patients who had continuous remission after induction with Tac and those assumed to have induced and maintained remission with IFX. Currently, many types of treatment options, such as anti‐TNF agents, anti‐α4β7 integrin antibodies, anti‐IL12/L23p40 antibody, and anti‐IL23p19 antibody and JAK inhibitors, are available for patients with UC in Japan. In the future, these therapies are likely to become more widely available, used earlier in the disease course, and for much longer periods. Appropriate use of existing time‐limited drugs, such as Tac, for moderate‐to‐severe UC patients should be considered, rather than the blind introduction of biologic therapy. However, the remission maintenance rate in patients receiving 5‐ASA and thiopurine after achieving remission with Tac was not high. The risk of relapse and reduced quality of life solely for the purpose of cost savings cannot be accepted. Therefore, additional strategies must be adopted. For example, patients with a history of thiopurine use should be recommended a biologic agent for reliable maintenance therapy. In addition, in cases in which remission is difficult to maintain with 5‐ASA and thiopurines alone, biomarkers should be regularly measured to detect relapse earlier and biologic agents should be initiated while the activity is mild.

Several limitations of the present study warrant mention. First, this was a retrospective study performed at a single center. Adverse events that were not recorded in the medical charts could not be collected. Second, the duration of Tac administration varied among patients. Although Tac is recommended to be discontinued approximately 3 months after the start of administration in Japan, most patients did not discontinue within 3 months because of their refractory disease course. Third, endoscopic remission may predict long‐term prognosis in refractory UC patients treated with Tac [5, 34], but biomarkers related to endoscopic remission, such as fecal calprotectin, fecal immunochemical test, or endoscopic scores, could not be examined in this study due to data loss. Finally, because there is no established regimen for patients refractory to Tac, additional treatment was chosen according to the decision of each doctor.

In conclusion, Tac should be chosen for thiopurine‐naïve patients with relatively short disease duration, even for patients with hypoalbuminemia and high CRP values at the start of Tac. Tac has the potential to induce remission even in patients with severe refractory UC and hypoalbuminemia, who are considered unfavorable candidates for IFX therapy. In addition, medical costs could be reduced by the appropriate use of Tac compared with biologics during the remission induction phase. Tac will continue to be a key drug for patients with severe refractory UC, even in the era of biologics.

## Ethics Statement

This study was approved by the Institutional Review Board of Okayama University Graduate School of Medicine and was a retrospective study of Tac for UC patients (IRB number: 2201‐003). Written informed consent from the patient was waived because the data were anonymously and retrospectively analyzed.

## Conflicts of Interest

The authors declare no conflicts of interest.

## Supporting information


**Data S1.** Supplementary Material

## Data Availability

All data required to evaluate the conclusions of this study are provided in the main text of the manuscript. This study did not include data from external repositories. Additional data related to this study were obtained from the authors. The datasets used and analyzed during the current study are available from the corresponding author upon reasonable request.
