# Supplementary material for: Induction Therapy With Oral Tacrolimus Provides Long‐Term Benefit in Thiopurine‐Naïve Refractory Ulcerative Colitis Patients Despite Low Serum Albumin Levels
Source: JGH Open. 2025 Apr 2;9(4):e70139. doi: 10.1002/jgh3.70139 (PMC11962645; doi:10.1002/jgh3.70139)
Supplement: Supplementary file 1 — Data S1. Supplementary Material [file JGH3-9-e70139-s001.docx]

| Drug | Dosage | Original Cost  (yen) | US$^†^ | Generic/Biosimilar  (yen) | US$^†^ |
| --- | --- | --- | --- | --- | --- |
| Tacrolimus | 1 mg | 390.0 | 2.49 | 153.5 | 0.98 |
| Azathioprine | 50 mg | 78.8 | 0.50 | — | — |
| 6-mercaptopurine | 1000 mg | 93.6 | 0.60 | — | — |
| pH-dependent mesalazine | 400 mg | 37.3 | 0.24 | 18.6 | 0.12 |
| time-dependent mesalazine | 500 mg | 51.8 | 0.33 | 8.0 | 0.05 |
| MMX time-dependent mesalazine | 1200 mg | 159.9 | 1.02 | — | — |
| Salazosulfapyridine | 500 mg | 9.3 | 0.06 | 6.5 | 0.04 |
| Infliximab | 100 mg | 54,950 | 350.31 | 20,727 | 132.14 |
| Adalimumab | 40 mg | 48,988 | 312.30 | 22,623 | 144.22 |
| Golimumab | 100 mg | 106,324 | 677.83 | — | — |
| Vedolizumab | 300 mg | 279,573 | 1782.31 | — | — |
| Ustekinumab,  intravenous infusion | 130 mg | 184,085 | 1173.56 | — | — |
| Ustekinumab, subcutaneous injection | 45 mg | 336,004 | 2142.06 | — | — |
| Tofacitinib | 5 mg | 2,260 | 14.41 | — | — |
| Apheresis (Adacolumn^R^) | 1 column | 118,000 | 752.26 | — | — |

**Supplemental Table. Prices for Calculating Cumulative Drug Costs**

† The exchange rate was US$1 = 156.85 yen in January 2025.


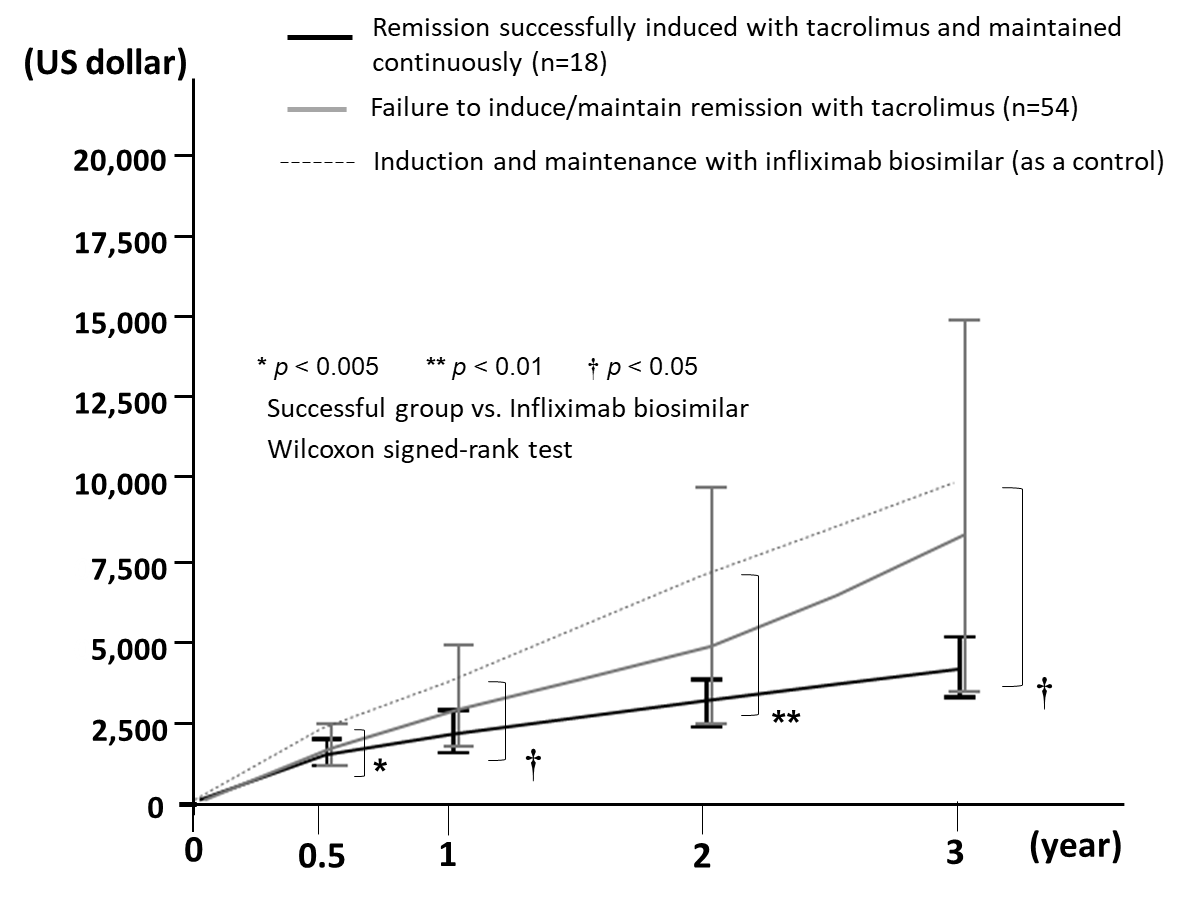


**Supplemental Figure. Cumulative cost over three years calculated using generic and biosimilar costs.**

The median cumulative drug costs over three years with Tac and the control case with IFX-biosimilar. The cumulative cost in patients with successful remission induction with Tac was significantly lower than those of the control patients with IFX at all time points (0-0.5; *p* < 0.005, 0-1; *p* < 0.05, 0-2; *p* < 0.01, and 0-3 years; *p* <0.05, Wilcoxon signed-rank test). For the group with Tac treatment, the upper horizontal line shows the 75th percentile, and the lower horizontal line shows the 25th percentile. The graph line connects the median.
